# Patient consent to publication and data sharing in industry and NIH-funded clinical trials

**DOI:** 10.1186/s13063-018-2651-2

**Published:** 2018-05-03

**Authors:** O’Mareen Spence, Richie Onwuchekwa Uba, Seongbin Shin, Peter Doshi

**Affiliations:** 1 University of Maryland School of Pharmacy, Baltimore, MD USA; 2 Notre Dame of Maryland University, Baltimore, MD USA

**Keywords:** Informed consent, Ethics, Responsible conduct of research, Publication bias, Reporting bias, Data sharing, Clinical trials, Clinical data

## Abstract

**Background:**

Participants are recruited into clinical trials under the assumption that the research will contribute to medical knowledge. Therefore, non-publication trials—and, more recently, lack of data sharing—are widely considered to violate the trust of trial participants. Existing practices regarding patient consent to publication and data sharing have not been evaluated. Analyzing informed consent forms (ICFs), we studied what trial participants were told regarding investigators’ intention to contribute to medical knowledge, publish trial results, and share de-identified trial data.

**Methods:**

We obtained 98 ICFs of industry-funded pre-marketing trials for all (17) antibiotics approved by the European Medicines Agency and 46 ICFs of publicly funded trials from the National Heart, Lung and Blood Institute Biologic Specimen and Data Repository Information Coordinating Center (BioLINCC) data repository. Three authors independently reviewed ICFs to identify and extract what was stated or implied regarding: (1) publication of results; (2) sharing de-identified data; (3) data ownership; (4) confidentiality of identifiable data; and (5) whether the trial will produce knowledge that offers public benefit. Consensus was obtained from the two reviewers with the greatest overall agreement on all five measures. Disagreements were resolved through discussion among all authors.

**Results:**

Four (3%) trials indicated a commitment to publish trial results; 140 (97%) did not commit to publishing trial results; six (4%) indicated a commitment to share de-identified data with third party researchers. Commitments to share were more common in publicly funded trials than industry-funded trials (7% vs 3%). A total of 103 (72%) ICFs indicated the trials will or may produce knowledge that offers public benefits, while 131 (91%) ICFs left unstated who “owned” trial data; of those with statements, the sponsor always claimed ownership. Patient confidentiality was guaranteed in 137 (95%) trials.

**Conclusions:**

Our results suggest that consent forms rarely disclose investigators’ intentions regarding the sharing of de-identified data or publication of trial results.

## Background

The non-publication of clinical trials has long been considered a violation of the trust of clinical trial participants who enrolled under an assumption of contributing to medical knowledge [1, 2] and in recent years, the research community has increasingly come to consider clinical trial data sharing to be another fundamental ethical obligation of trialists [3, 4]. These ethical norms are supported by survey data which show that people enroll in clinical trials with an assumption of altruism—that their participation will benefit others [5–7]. However, to our knowledge, an empirical evaluation of actual practices regarding patient consent to publication and data sharing has not been carried out.

Informed consent forms (ICFs) are generally the closest to a “written contract” between trial investigators and research study participants. They allow for a deeper understanding of the a priori intentions of investigators, at least insofar as what they consider part of their obligations to trial participants. But ICFs themselves have largely escaped being the focus of comparative studies because ICFs are generally treated as confidential by investigators, institutional review boards, and regulators.

In this study, our objective was to determine, using ICFs, what trial participants were told regarding investigators’ intention to contribute to medical knowledge, publish trial results, and share de-identified trial data. We also investigated statements regarding maintaining patient confidentiality and data ownership.

## Methods

We made freedom of information requests to the European Medicines Agency (EMA) for ICFs from all industry sponsored pre-marketing clinical trials of all approved antibiotics. To compare industry-funded trials with publicly funded trials, we searched for ICFs from all clinical trials listed in the National Heart, Lung and Blood Institute (NHLBI) Biologic Specimen and Data Repository Information Coordinating Center (BioLINCC) [8]. The NHLBI repository was established in 2000 and includes patient-level data and trial documents, such as trial protocols and ICFs, for over 100 clinical trials [9]. We also contacted the BioLINCC staff directly to obtain any ICFs that might be available but not downloadable via the website. As ICFs are generally not publicly accessible, our sample of ICFs was chosen for pragmatic reasons of data availability.

We included all clinical trials with available ICFs. We excluded trials if the ICF was not available, not for the main clinical trial (e.g. the only ICF available was for a telephone survey occurring alongside the main clinical trial), or incomplete. If multiple ICFs were available, we chose the most recent ICF dated before the start of patient enrollment. If identically dated ICFs existed for differently aged populations, we chose the form used with the oldest population. When multiple ICFs met these criteria, for example multiple ICFs for different study sites, we chose the first ICF that appeared in the documents.

Three authors independently reviewed ICFs to identify and extract what was stated or implied regarding: (1) publication of results (e.g. in a journal article, trials registry, or other publication/report that would be publicly available); (2) sharing de-identified data (i.e. information sufficient for re-analysis by researchers); (3) data ownership (i.e. property); (4) confidentiality of identifiable data; and (5) whether the trial will produce knowledge that offers public benefit (i.e. public benefit beyond the potential product itself). Consensus was obtained from the two reviewers with the greatest overall agreement on all five measures. Disagreements were resolved through discussion among all authors.

## Results

As of January 2017, we obtained documents from EMA for 134 clinical trials of 17 antibiotics (retapamulin, inhaled aztreonam, inhaled colistin, daptomycin, fidaxomicin, doripenem, ertapenem, telithromycin, inhaled tobramycin, trovafloxacin, tigecycline, telavancin, ceftaroline, dalbavancin, oritavancin, tedizolid, bedaquiline). We excluded 36 trials (34 had no ICF available, one had only an incomplete ICF, and one ICF was not for the primary clinical trial). A total of 118 publicly funded clinical trials were available through BIOLINCC and the NHLBI Data Repository staff. We excluded 17 because a protocol was not available. Of the remainder, we excluded 52 with no ICF available, two where the ICF was not for the primary study, and one with an incomplete ICF. The final sample included 144 ICFs (98 industry-funded trials, 46 publicly funded trials), enrolling 224,315 participants between 1983 and 2013.

Of the ICFs, 140 (97%) did not indicate a commitment to publish trial results. The remaining ICFs reported a commitment to publish (1% industry-funded trials; 7% publicly funded trials); 121 (84%) did not indicate any intention to share de-identified data with third-party researchers; six (4%) committed to sharing de-identified data; and 14 (10%) indicated de-identified data may be shared. Commitments to share were more common in publicly funded trials than industry-funded trials (7% vs 3%). Similarly, 17% of publicly funded trials reported data may be shared, compared to 6% of industry-funded trials (Table 1).

**Table 1:** Characteristics of trials and ICFs position on publication, data sharing, data ownership, confidentiality, and public benefit

	Total (*n* = 144)	Industry-funded trials (*n* = 98)	Publicly funded trials (*n* = 46)
Characteristics of trials			
Year of participant enrollment commencement, median (range)	2002 (1983–2013)	2003 (1994–2013)	1995 (1983–2012)
Number of participants, median (total)	460 (224,315)	319 (35,181)	914 (189,134)
ICFs’ position (n, %)			
Publication of results			
Indicated a commitment to publish	4 (3)	1 (1)	3 (7)
Indicated a commitment not to publish	0 (0)	0 (0)	0 (0)
Did not indicate a commitment to publish	140 (97)	97 (99)	43 (93)
Sharing de-identified data with third-party researchers			
Indicated a commitment to share	6 (4)	3 (3)	3 (7)
Indicated a commitment not to share	3 (2)	2 (2)	1 (2)
Indicated de-identified data may be shared	14 (10)	6 (6)	8 (17)
Did not indicate an intention to share	121 (84)	87 (89)	34 (74)
Will the trial produce knowledge that offers public benefit?			
Will produce knowledge	33 (23)	15 (15)	18 (39)
May produce knowledge	70 (49)	51 (52)	19 (41)
Unclear statement	1 (1)	1 (1)	0 (0)
No statement available	40 (28)	31 (32)	9 (20)
Explicit statement of data ownership			
Yes - sponsor	13 (9)^a^	10 (10)^a^	3 (7)^a^
Yes - other party	0 (0)	0 (0)	0 (0)
Yes - trialist	0 (0)	0 (0)	0 (0)
Yes - participants	0 (0)	0 (0)	0 (0)
Cannot determine or no statement available	131 (91)	88 (90)	43 (93)
Are patients provided with a general guarantee of confidentiality?			
Confidentiality is guaranteed	137 (95)	93 (95)	44 (96)
Unclear statement	5 (4)	4 (4)	1 (2)
No statement available	2 (1)	1 (1)	1 (2)

^a^In three of ten industry-funded trials and three of three publicly funded trials, ownership referred to ownership of biological samples only

One hundred and three (72%) ICFs indicated the trials will or may produce knowledge that offers public benefits. This was most often reported in publicly funded trials than industry-funded trials (80% vs 67%). Forty (28%) did not include a statement regarding the production of knowledge and 131 (91%) ICFs left unstated who “owned” trial data; of those with statements, the sponsor always claimed ownership. Patient confidentiality was guaranteed in 137 (95%) trials (Table 1).

Table 2 shows verbatim examples of language used to express intentions regarding data sharing, publication and ownership. We classified words such as “require” or “will” to indicate a clear commitment to publish (or share data). We classified language such as “may,” on the other hand, as indicating a lack of clear commitment, as we considered the phrase “data may be published or shared” as logically equivalent to “may not be published or shared.”

**Table 2:** Selected examples of consent form language on publication, data sharing, and data ownership

Sponsor indicated a commitment to publish	
“When the results of this study are published, no data will be listed by name or ID number.” (CAMP trial, publicly funded)	
“When the results of this study are made public, the doctors will not use your name or let anyone know about you personally.” (OAT 1 trial, publicly funded)	
Sponsor did not indicate a commitment to publish	
“The Study Team may also use my information to prepare reports or publications about the study.” (DUR001–104 trial, industry-funded)	
“If any publication or presentations result from this study, you will not be identified by name.” (ROMICAT 2 trial, publicly funded)	
Sponsor indicated a commitment to share	
“The National Heart, Lung, Blood Institute (NHLBI) requires that the data collected during a research study is made available to qualified investigators and non-study researchers.” (HF Action trial, publicly funded)	
“Data collected from you and other participants in this study will be shared with other doctors in the research field, but no names of patients will be used.” (CP-AI-005, industry-funded)	
Sponsor indicated de-identified data may be shared	
“Other researchers who are approved through standard, approved agreements may be permitted to analyze the data without your personal identifying information.” (COAG trial, publicly funded)	
“Study data may be published or shared with other researchers, but the identity and medical information of each study participant will remain strictly confidential.” (TBM100C 2302 trial, industry-funded)	
Explicit statement of data or biological sample ownership - Sponsor	
“Data collected and recorded on study forms are the property of Corus Pharma, Inc.” (CP-AI-006 trial, industry-funded)	
“All research samples will become property of the NHLBI after conclusion of the BMT CTN Protocol #0102 study” (BMT-CTN-0102 trial, publicly funded)	

## Discussion

Our results suggest that investigators generally have not considered their trial publication and data sharing intentions to be part of the written informed consent process. Of ICFs, 97% did not contain any statements indicating a commitment to trial publication. Of the 3% that did, in all cases, the commitment was implicit, appearing in passages of ICFs intended to clarify patient privacy protections, not provide definitive statements about trial publication intentions.

By contrast, investigators routinely, 72% of ICFs, informed study participants that the trial will or may produce knowledge that offers public benefit. This is consistent with the notion that clinical research aims to produce generalizable knowledge [10] and also conforming to patients’ altruism.

Although we did not ascertain the publication status of trials in our study, empirical research has indicated, for both public and industry-funded trials, that around half of trials are published [11–15]. This suggests that trials are being published despite a lack of written commitment in ICFs. Similarly, while the majority (84%) of ICFs said nothing about data sharing, our study included all publicly funded trials which are currently sharing de-identified data through BioLINCC.

While investigators never indicated a commitment not to publish their trial, in three (2%) ICFs, investigators did in fact indicate a commitment not to share data. This finding is important because industry [16] and academic [17] authors have argued that in specific instances, data sharing would violate the written informed consent of trial participants. Our finding confirms that such guarantees do occur but are rare.

The ethical importance of trial publication and data sharing is emphasized in foundational documents such as the Declaration of Helsinki which since 2000 has stated that investigators have an “ethical obligation” to ensure study “results should be published or otherwise publicly available” [18]. But our results raise questions about the best mechanism for meeting this obligation. ICFs have been widely criticized for being overly long, complex, and difficult to read [19], and on these grounds, some may argue against including information describing investigators’ publication and data sharing intentions in ICFs.

However, at least for data sharing, proactive disclosure of the investigators’ intentions in ICFs can be expected to become far more common following passage of the revised (2017) Common Rule that now requires explicit disclosure that data sharing can take place (unless investigators explicitly commit not to share data) [20]. This rule appears compatible with survey data that suggests that while a minority of patients oppose de-identified data sharing, the large majority believe that disclosing potential data sharing is important during the informed consent process [21]. The revised Common Rule, however, does not contain any requirements regarding disclosure of investigators’ intentions to publish the clinical trial results.

This study does little to resolve the question that has been posed for at least two decades: “whose [trial] data are they anyway?” [22]. Of ICFs, 91% did not say anything about data ownership, and of those that did, they universally stated that sponsors/investigators owned the data.

Our study has limitations. We included ICFs that were dated before patient enrollment and therefore any subsequent changes to the ICF were not analyzed. Also, because we only had access to a full cohort of industry-funded trials for antibiotics and ICFs that were available for publicly funded trials at one National Institute of Health (NIH) agency, our findings may not be generalizable to other therapeutic areas or all privately and publicly funded trials.

## Conclusions

While investigators may intend to publish and even share data, their intentions are not often made clear to participants in ICFs. This may change, with respect to data sharing, following the passage of the revised Common Rule.

## Abbreviations

BioLINCC: Biologic Specimen and Data Repository Information Coordinating Center
EMA: European Medicines Agency
ICF: Informed consent form
NHLBI: National Heart, Lung and Blood Institute
NIH: National Institute of Health
